# A blockchain-based certifiable anonymous E-taxing protocol

**DOI:** 10.1371/journal.pone.0270454

**Published:** 2022-07-05

**Authors:** Huimin Niu, Ting Li, Xiugang Gong

**Affiliations:** 1 Research Direction for Information Security, School of Computer Science and Technology, Shandong University of Technology, Zibo, Shandong, China; 2 Research Direction for Data Mining, School of Computer Science and Technology, Shandong University of Technology, Zibo, Shandong, China; 3 Research Direction for Embedded Systems, Detection and Control Technology, School of Computer Science and Technology, Shandong University of Technology, Zibo, Shandong, China; University College of Engineering Tindivanam, INDIA

## Abstract

The security of the tax system is directly related to the development of a country. The conventional process of tax payment laborious steps, so this process becomes a cause of irregularities among taxpayers and tax authorities, increasing the rate of corruption in tax collection. Blockchain, as a distributed ledger technology, its unique advantages and promising applications in taxation offer an effective solution to the problems of electronic taxation. However, the transparency of blockchain exists the risk of privacy disclosure, the high degree of anonymity brings the problem of lack of user supervision. Therefore, for balancing the contradiction of taxpayer privacy and supervision, we propose a blockchain-based self-certified and anonymous e-taxing scheme, which uses blockchain as the underlying support, and utilizes cryptography technology such as self-certified public key, Diffie-Hellman, to reduce the taxpayer′s reliance on the certificate authority, and protects the taxpayer′s anonymity while realizing the tracking of the real identity of malicious taxpayers. The security analysis proves that the scheme has the properties such as anonymity, conditional privacy and unforgeability, etc. Finally, performance analysis shows that compared with similar schemes, the scheme significantly improves the registration efficiency, proving its practicability and implementability.

## Introduction

At present, in many countries, taxation is an important avenue for governments to raise funds to finance their projects and programmes [1]. It has become a major source of public revenue, and its healthy and stable development plays an important role in the macroeconomic regulation of the country. Consequently, providing a secured system should be our first priority. Traditional paper tax is not only tedious and inefficient, but also cannot efficiently realize the cross-territory and cross-space taxation mode. Over the years, with the development of the economy and society and the diversification of market subjects, the mode of tax collection and management keeps pace with the times. Governments have invested a lot of resources to replace the traditional paper tax collection model by adopting electronic filing, which has not only improved tax collection efficiency but also facilitated tax compliance [2]. However, with the diversification of taxpayer types, multidimensional demand and business diversification, tax collection has also brought new challenges: the opaqueness of data worsens the asymmetry of tax information; over-reliance on centralization makes it difficult to trace the nature of things in real time across regions and subjects. If tax authorities and taxpayers do not trust each other in handling data, it will be more difficult to implement a centrally administered tax system. In particular, a recent report shows that the European Union lost 152 billion euros only in 2015 due to inadequate tax collection systems (https://news.fx678.com/201709280602501445.shtml). In recent years, some multinational companies have taken advantage of the differences in tax rates between countries to evade tax, with Alphabet′s Google transferring 15.9 billion to Bermuda Shell companies in 2016, successfully avoiding $1 billion in taxes (https://www.yicai.com/news/5388587.html). Therefore, we urgently need a more complex, efficient and scientific tax system.

With the advent of the era of big data, blockchain technology came into being and attracted wide attention from various countries. Various countries began to study how to use blockchain technology to accelerate the pace of tax collection. Blockchain is not only a new carrier to improve tax efficiency and quality, but also provides new ideas for the future development of tax collection and management. Blockchain, as a distributed database, has the characteristics of anonymity, consensus, traceability, transparency, decentralization [3]. It fits perfectly with the tax system and provides new ideas for improving tax collection efficiency, informatization of taxation and perfecting the taxation system. The specific applicability is as follows: the distributed database solves the problems of data storage space limitation and data island between different systems; the decentralization of blockchain realizes tax information sharing, overcomes the problems of difficult verification of tax information and weakening of single centralized storage; the traceability and non-tamperability of transaction data solves the problems of data dispersion and difficult query, and helps to establish an open and transparent tax database.

Blockchain-based e-tax applications are currently receiving widespread attention. In [4–6], blockchain technology has been employed to create decentralized applications that track value-added tax(VAT) transactions of businesses, which can not only effectively track whether and where VAT has been paid, but also reduce tax compliance costs for businesses and individuals, improve taxpayer compliance and the ability of tax authorities to supervise micro-transactions. Moreover, Saragih and Setyowati [7] discuss the benefits of blockchain in the tax administration, and the factors affecting blockchain technology in tax administration. In the tax system applied by blockchain technology, it provides effective solutions for the problems existing in e-tax, such as tax information disclosure, tax collection and tax service. Demirhan [8] proposes to create smart contracts with different types of tax algorithms in a blockchain-based tax model, which can coordinate records between multiple parties in real time and automatically, prevent inefficient tax operations and reduce or prevent fraud among parties involved in the management chain in e-tax. Tasca et al. [9] also mention that smart contracts in blockchain technology enable the validation and automation of tax returns, significantly reducing the risk of tax avoidance, fraud and evasion. Currently, many people consider that security, privacy, costs, and regulatory issues are the greatest challenges acing the current information age [10]. Preserving user privacy is a critical issue when it comes to collecting and handling highly sensitive personal data [11]. Many academics have discussed how to use blockchain to protect user privacy in various scenarios, for example, healthcare [12, 13], vehicular ad hoc networks(VANETs), e-ticketing, etc. However, while the anonymity of blockchain protects user privacy, it also provides an umbrella for some illegal and criminal acts. For example, in blockchain-based electronic tax applications, it is unable to track illegal transactions by linking the transaction records to the relevant traders, which makes auditing difficult and tax evasion cannot be detected and stopped in time. The open and transparent function allows any node on the network to view and supervise the tax information on the blockchain. Although it solves the problem of difficult and slow detection of counterfeit tickets, there may be unscrupulous elements to infer the taxpayer′s wallet address, identity information and lifestyle habits by analysing the tax pattern [14]. Security is the most significant one where the user′s details are highly confidential from both legal and ethical sides. Article 34 of Act No. 28 of 2007 on general provisions and procedures for taxation sets out the importance of keeping tax data confidential [7]. The provisions described in the Act show that the security of taxpayers’ data is crucial and should be seriously considered.

### Our contribution

Based on the aforementioned challenges, the main contributions of this paper are summarized as follows.

1. We propose a blockchain-based self-certified and traceable e-taxing scheme that verifies the authenticity of taxpayers without revealing their true identity, thus balancing the contradiction of taxpayer privacy and supervision.
2. We provide a conditional privacy protection, i.e. certificate authority can track misbehaving taxpayers in e-tax and revoke the true identity of misbehaving taxpayers from causing any further damage.
3. We propose an efficient self-certified scheme that self-certified public key [15] system instead of the certificate-based public key system. The scheme not only reduces the amount of public key storage and computation, improves the registration efficiency of the system, but also reduces the security risk by reducing the dependence on the certificate authority, so that the scheme has higher security.

### Organization

The remainder of this paper is organized as follows. First is an introduction that the necessary preparations. Next, we describes the related work, the system model and security requirements. This is followed by construction of the scheme. The next section describes the security analysis of the protocol. The conclusions of protocol are presented at the end of the article.

## Related works

The lack of privacy protection and data leakage will raise many problems for blockchain-based e-tax systems. Some scholars have also taken measures to improve the privacy of taxpayers. Considering that efficient tax models require a trade-off between privacy and transparency, Hoffman et al. [16] propose a blockchain that can implement an access control policy by deploying a set of global smart contracts on a federal ledger managed on the chain, defining each node′s role and access to data. This policy not only solves a single point of failure for the entire system, but also avoids errors and delays in processing tax data on a global scale. Fatz et al. [17] propose a conceptual design of confidentiality and distributed tax document exchange system, stating that zero-knowledge proofs solves the dilemma between transparency and confidentiality in tax systems. Magdalena [18] achieved taxpayer anonymity by adding a serial number to the e-ticket, thus avoiding the reuse of tax slips by malicious users and tying the taxpayer′s identity information to the tax slip to prove its uniqueness. Li and Niu [19] established a federated block-based chain-based e-ticketing system that not only uses ring signatures to achieve anonymity in a hybrid currency protocol, but also guarantees the unforgeability of tickets through multiple signatures.

Although the above scheme improve the protection of user privacy, it does not consider how to balance the contradiction between anonymity and accountability, and still face the challenge of difficult supervision. However, in the existing work, some scholars have discussed the application of balancing anonymity and traceability in other scenarios, such as, wireless body area networks(WBANs) [20], roaming service [21]. In addition, in [22] proposed conditional tracking mechanisms for VANETs, and used an efficient anonymous two-way authentication scheme. In addition, in [23] proposed an anonymous authentication scheme for wireless body area networks based on low-entropy password, which proved its security in the random oracle model. In [24] through secure authentication code transfer between the consecutive roadside unit. In fact, these anonymity schemes are based on the Diffie-Hellman problem under discrete logarithms, whereas the security of our scheme is based on the Diffie-Hellman assumption under elliptic curves, which is much more difficult than the Discrete Logarithm Problem over Finite Fields [25]. Furthermore, different from [23], the security proof in our scheme on anonymity is under the generic group model.

## Preliminaries

### One-Way Hash Function Assumption(OWHF)

Let *H*(⋅) be a one-way hash function [26]. We assume that the input of the hash function is randomly and uniformly distributed, and the output is also randomly and uniformly distributed. For given a arbitrary message *M*, it is easy to calculate *H*(*M*). The following tasks are computationally infeasible:

1. Given an integer *H*(*M*), it is infeasible to calculate *M*.
2. Given an integer *H*(*M*), it is infeasible to find another message *M*′ satisfying that *H*(*M*) = *H*(*M*′).

### Elliptic Curve Diffie-Hellman (ECDH)

It is a simulation of Diffie-Hellman [27] key change in a finite field and based on Elliptic Curve Discrete Logarithm Problem(ECDLP). In elliptic curve the public parameters *P* as a generate in group *G*, given any point (*P*, *aP*, *bP*)∈*G*, *a*, *b* ∈ [1, *n* − 1]. It is difficult to compute *abP*. The advantage of adversary A in solving ECDH problem is defined as:

$$\begin{array}{c} Adv_{A}^{ECDH}\left(\lambda \right)=Pr\left[abP\leftarrow \left(aP,bP\right)\right] \end{array}$$

For any polynomial-time, no adversary A can solve the ECDH problem with non-negligible advantage.

### Non-interactive zero-knowledge proofs

Non-interactive Zero-knowledge Proofs(NIZK) [28] is a delicate cryptographic protocol, which usually studied in the common reference string (CRS) model.

Let (*ω*, *x*)∈*R* be a binary relation, where *x* is a common reference string and *ω* is a witness for *x*. A prover to generate a proof and convince the verifier that he indeed knows a certain quantity *ω* satisfying (*ω*, *x*)∈*R* without leaking any additional knowledge of the secret. Informally, the NIZK satisfies the following properties [29].

- *Completeness*. A prover can generate a proof such that it can be passed through the verification by the verifier with probability 1.
- *Computational soundness*. No polynomial-time adversary is capable of forging a valid attestation that can be accepted by the verifier ith non-negligible probability.
- *Zero-knowledge*. The procedure only reveals the statement rather than any secret.

### Smart contact

Smart contacts are automatically stored and executed in the blockchain as part of a transaction, which has a better security system than the traditional paper contacts [30]. The necessary fairness and credibility can be ensured directly through the performed partially or fully self-executing of contractual clauses.

In [31], it is discussed that the new data capabilities and possibilities of smart contract applications in tax management. In the blockchain distributed network [32], each node, i.e., the tax authority, deploys the relevant smart contract and publishes the taxpayer′s tax payment information. Then the user executes the smart contract to know the amount of tax to be paid. All the execution results are recorded as a transaction, which is irreversible and traceable. Meanwhile, each node will update the duplicate locally based on the current execution result after running the smart contract. Its secure distributed environment makes smart contract widely used in practice.

## System model and security requirements

In this section, we describe the system model and system components of the blockchain-based certifiable anonymous e-taxing protocol. Then we introduce the related security requirements.

### System model

**Fig 1:**
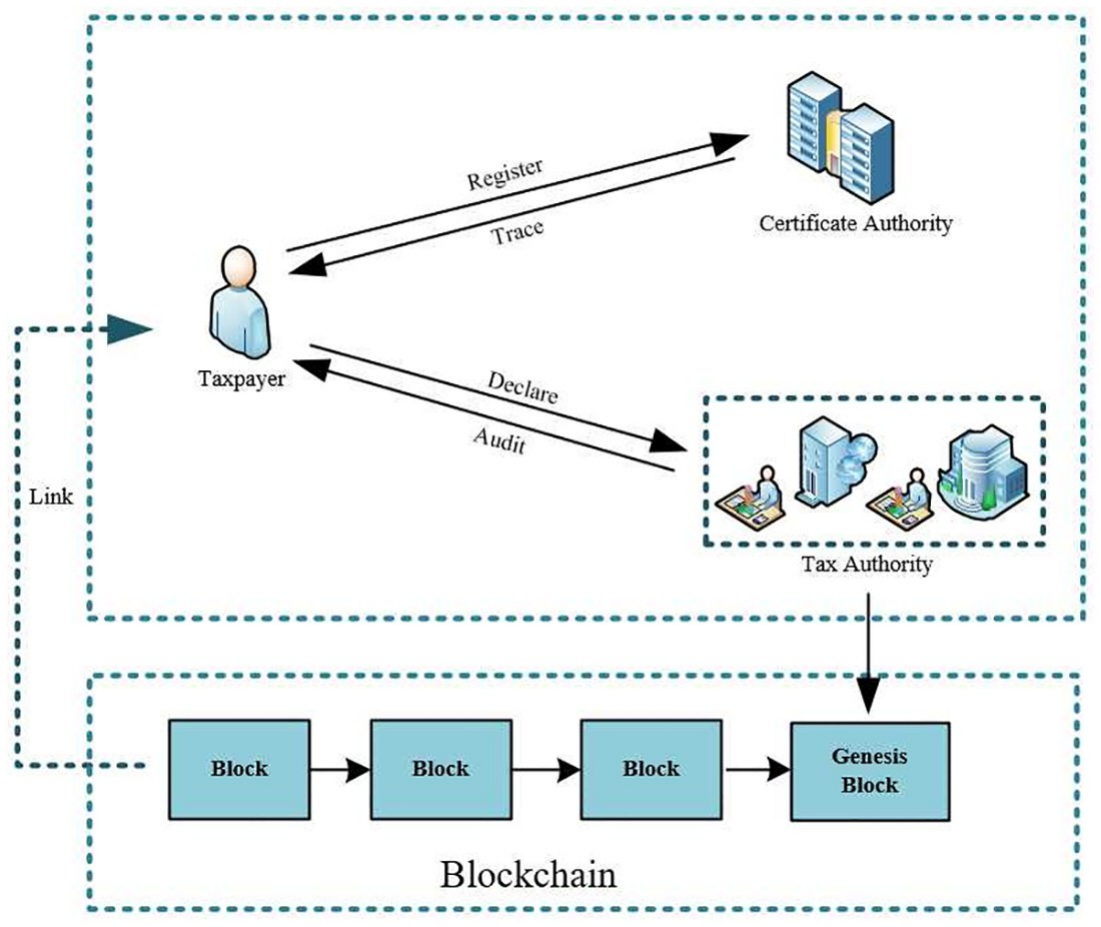
The framework of the blockchain-based certifiable anonymous e-taxing system.

As shown in Fig 1, four entities are involved in our system, namely, the certificate authority, the tax authority, the taxpayer and the smart contract.

- *Certificate Authority*, identified by *CA*, is mainly to initialization of system parameters. Moreover, *CA* is responsible for issuing certificates and maintaining the list of eligible registrations, authenticating and managing taxpayers’ identity, and revealing the true identity of misbehaved taxpayers.
- *Tax Authority*, identified by *TA*, deploys smart contracts and publishes the tax payment information of the corresponding taxpayer to smart contracts.
- *Taxpayer*, identified by *TU*, registers with *CA* to become a legal taxpayer and executes the smart contract to know the tax amount.
- *Smart Contract*, identified by *SC*, resemble the third-parties (e.g. brokers) involved in a deal, ensuring trust among the parties. Specify detailed rules for each role based on pre-determined set of conditions.

### System components

A blockchain-based certifiable anonymity e-taxing protocol consists of the following polynomial-time algorithms *Setup*, *Register*, *Declare*, *Audit and Trace*.

- **Setup**(λ) → (*PP*, (*P*_*CA*_, *S*_*CA*_)(*P*_*TA*_, *S*_*TA*_)). The setup algorithm is a function that takes as input a security parameter λ, and outputs the system public parameters *PP*, *CA*′s key pair (*P*_*CA*_, *S*_*CA*_) and *TA*′s key pair (*P*_*TA*_, *S*_*TA*_).
-
$\text{Register}\left(PP,\left(P_{CA},S_{CA}\right),RID_{i}\right)\to \left(ID_{i},S_{TU_{i}},c_{i}\right)$ . This is an interactive protocol between a taxpayer and *CA*, which takes the system public parameter *PP*, a taxpayer′s real identifier *RID*_*i*_, *CA*′s key pair (*P*_*CA*_, *S*_*CA*_) as input, and outputs the taxpayer′s pseudoidentity *ID*_*i*_(i.e. tax identification number), private key $S_{TU_{i}}$ and self-certified key *c*_*i*_.
-
$Declare\left(PP,S_{TU_{i}},P_{TA},ID_{i},M_{i},T_{i}\right)\to \left(\sigma_{i},C_{i}\right)$ . This is an interactive protocol between a taxpayer and *TA*, which takes the system public parameter *PP*, taxpayer′s private key $S_{TU_{i}}$, tax authority′s public key, *P*_*TA*_ taxpayer′s pseudoidentity *ID*_*i*_, the tax return *M*_*i*_ ∈ {0, 1}* and timestamp *T*_*i*_. It output a signature *σ*_*i*_ and ciphertext *C*_*i*_.
- **Audit**(*PP*, *P*_*CA*_, *ID*_*i*_, *T*_*i*_, *c*_*i*_, *C*_*i*_, *σ*_*i*_) → (0/1). The algorithms takes the system public parameter *PP*, the *CA*′s public key *P*_*CA*_, the taxpayer′s *ID*_*i*_, timestamp *T*_*i*_ and its self-certified key *c*_*i*_, signature *σ*_*i*_, ciphertext *C*_*i*_ as input. It outputs 1 if the tuple is valid and 0 otherwise.
- **Trace**(*PP*, *S*_*CA*_, *ID*_*i*_) → *RID*_*i*_. This algorithm is performed by *CA*. It takes the system public parameter *PP*, *CA*′s private key *S*_*CA*_, and the taxpayer′s pseudoidentity *ID*_*i*_ as input. It outputs the corresponding a malicious taxpayer′s real identifier *RID*_*i*_.

### Security requirements

A blockchain-based certifiable anonymous e-taxing protocol requires the following properties.

- *Anonymity*. Taxpayers should be kept anonymous when paying taxes, and no one can link a tax return to a true identity of taxpayer.
- *Unforgeability*. No one can forge taxpayer′s certificate and signature, only certified taxpayers can generate a tax return correctly.
- *Traceability*. When a illegal tax return is found, the misbehaved taxpayer′s identity can be tracked and exposed by the certificate authority.

#### Anonymity

Anonymity of a blockchain-based certifiable anonymous e-taxing protocol is an essential security property. Given a tax identification number, no adversary except *CA* could associate the true identity of the taxpayer with the tax identification number with non-negligible probability.

Anonymity for e-taxing schemes is defined as the following game between the Challenger C and the Adversary A. A is given access to an *register oracle*. Here A is functional in two phases, a choose phase and a guess phase.

**Definition 1 (Anonymity).** A blockchain-based certifiable anonymous e-taxing protocol satisfies anonymity if for any polynomial-time adversary A, its advantage $Adv_{A}^{Anony}\left(\lambda \right)$ is negligible in winning the following game.

${\text{EXP}}_{A}^{Anony}\left(\lambda \right)$

1. (*PP*, (*P*_*CA*_, *S*_*CA*_), (*P*_*TA*_, *S*_*TA*_)) ← *Setup*(λ)
2. **Choose**
$$\begin{array}{c} \left(ID_{1},ID_{2}\right)\leftarrow A^{O_{reg}\left(S_{CA},\cdot ,\cdot \right)}\left(choose,PP,RID\right); \end{array}$$
$$\begin{array}{c} j\in \left\{0,1\right\};\;\left(A_{j},ID_{j}\right)\leftarrow Registe\left(PP,RID_{j}\right) \end{array}$$
3. **Guess**
$$\begin{array}{c} j^{\prime }\leftarrow A\left(guess,A_{j},ID_{j}\right) \end{array}$$
4.
A wins if *j*′ = *j*. We denote by
  $$\begin{array}{c} Adv_{A}^{Anony}\left(\lambda \right)=\left|Pr\left[A\;wins\;the\;game\right]-\frac{1}{2}\right| \end{array}$$

The *ID*_1_, *ID*_2_ were not queried to *register oracle* in the choose stage.

#### Unforgeability

We now provide a rigorous definition of security by defining the *Unforgeability, Experiment*, which requires that no adversary can forge a valid signature, even if it obtain one or more certified address by compromise the *CA*/Taxpayer.

Unforgeability for e-taxing schemes is defined as the following game between the Challenger C and the Adversary A. In this game, our definition is adaptive and allow the adversary to adaptively choose a tax return existing in the forgery. A is given access to an *register oracle* and *a sign oracle*. Here A is functional in two phases, a choose phase and a guess phase.

**Definition 2 (Unforgeability).** A blockchain-based certifiable anonymous e-taxing protocol satisfies Unforgeability if for any polynomial-time adversary A, its advantage $Adv_{A}^{Unfo}\left(\lambda \right)$ is negligible in winning the following game.

${\text{EXP}}_{A}^{Unfo}\left(\lambda \right)$

1. (*PP*, (*P*_*CA*_, *S*_*CA*_), (*P*_*TA*_, *S*_*TA*_)) ← *Setup*(λ), *and set L* ← ∅, *S* ← ∅
2. **Choose**
$$\begin{array}{c} \left(ID_{i},c_{i}\right)\leftarrow A^{O_{reg}\left(S_{CA},\cdot ,\cdot \right)}\left(choose,PP,RID\right);\;Update\;L\leftarrow L⋃c_{i}; \end{array}$$
$$\begin{array}{c} \sigma_{i}\leftarrow A^{O_{sign}\left(S_{TU_{i}},\cdot ,\cdot \right)}\left(choose,PP,c_{i},M_{i}\right);\;Update\;S\leftarrow S⋃\left(c_{i},M_{i},\sigma_{i}\right) \end{array}$$
3. **Guess**
$$\begin{array}{c} \sigma^{*}\leftarrow A\left(guess,M^{*},c^{*}\right) \end{array}$$
4.
A wins if
  $$\begin{array}{c} Verify_{y}^{ECDSA}\left(M^{*},\sigma^{*}\right)=1\;and\;c^{*}\notin L,\;and\;\left(c^{*},M^{*},\sigma^{*}\right)\notin S \end{array}$$
  We denote by
  $$\begin{array}{c} Adv_{A}^{Unfo}\left(\lambda \right)=Pr\left[A\;wins\;the\;game\right] \end{array}$$

#### Traceability

Traceability for the proposed protocol is also a core security requirement, this ensures that even if all tax authority and malicious taxpayer collude, they cannot produce a signature that traces to an honest taxpayer whose personal secret key has not been learned by the adversary.

Traceability for e-taxing schemes is defined as the following game between the Challenger C and the Adversary A. A is given access to an *register oracle* and *a sign oracle*. Here A is functional in two phases, a choose phase and a guess phase. A corrupts a set Co of taxpayers adaptively.

**Definition 3 (Traceability).** A blockchain-based traceable certified e-taxing protocol satisfies traceability if for any polynomial-time adversary A, its advantage $Adv_{A}^{Trace}\left(\lambda \right)$ is negligible in winning the following game.

${\text{EXP}}_{A}^{Trace}\left(\lambda \right)$

1. (*PP*, (*PCA*, *SCA*), (*PTA*, *STA*)) ← *Setup*(λ), *and set Co* ← ∅.
2. **Choose**
$$\begin{array}{c} \left(ID_{i},c_{i}\right)\leftarrow A^{O_{reg}\left(S_{CA},\cdot ,\cdot \right)O_{sign}\left(S_{TU_{i}},c_{i},\cdot ,\cdot \right)}\left(choose,PP,S_{CA},S_{TU_{i}},c_{i}\right);\\ \text{Update}\;\text{Co}\leftarrow \text{Co}⋃(ID_{i},c_{i}) \end{array}$$
3. **Guess**
$$\begin{array}{c} \left(M^{{}^{\prime }},\sigma^{{}^{\prime }}\right)\leftarrow A^{O_{sign}\left(S_{TU_{i}},c_{i},\cdot ,\cdot \right)}\left(guess,PP,c^{{}^{\prime }},ID^{{}^{\prime }}\right) \end{array}$$
4.
A wins if
  $$\begin{array}{c} Verify_{y}^{ECDSA}\left(M^{\prime },\sigma^{\prime }\right)=0;\;\left(c^{\prime },ID^{\prime }\right)\notin Co \end{array}$$
  $$\begin{array}{c} Trace(PP,S_{CA},\sigma^{\prime })=⊥ \end{array}$$
  We denote by
  $$\begin{array}{c} Adv_{A}^{Trace}\left(\lambda \right)=Pr\left[A\;wins\;the\;game\right] \end{array}$$

## The proposed scheme

In this section, we introduce the concrete construction of a blockchain-based certifiable anonymous e-taxing protocol. The protocol consists of five parts: *Setup, Register, Declare, Audit and Trace*.

**Fig 2:**
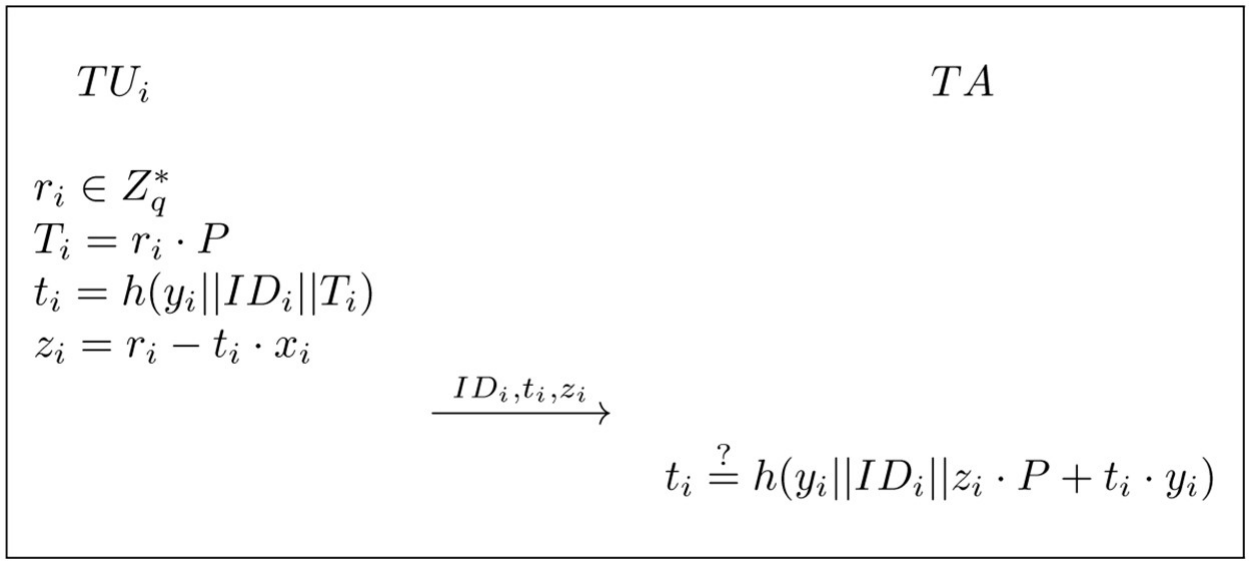
Zero-konwledge proof for legal identity.

***A. System initialization*** To setup the tax system, *CA* initializes the system parameters and generates his public/private key pair, and *TA* deploys the smart contract on the blockchain. Specifically, the following steps are executed.
  - Pick a random λ as the security parameter, and *M* ∈ {0, 1}* as a identifier of the tax return.
  - *CA* choose a cyclic group *G*_1_ with prime order *q*, where *G*_1_ is generated by *P*. *TA* chooses an elliptic curve *E* defined over *Z*_*p*_ where *p* is a prime. Let *G* ∈ *E*(*Z*_*p*_) be a base point of order *n* which is a prime. The reduction function be some function *f*: < *G* > → [0, *n* − 1], and *f*(*R*) = *x*_*R*_ *modn* where *x*_*R*_ is an integer representation of the *x*-coordinate of the elliptic curve point *R*.
  - *CA* selects $S_{CA}\in Z_{q}^{*}$ at random as *CA*′s private key, and computes *P*_*CA*_ = *S*_*CA*_ ⋅ *P* as *CA*′s public key. *TA* selects chooses $S_{TA}\in Z_{q}^{*}$ as *TA*′s private key, and computes *P*_*TA*_ = *S*_*TA*_ ⋅ *P* as *TA*′s public key.
  - *CA* chooses a secure hash function *h*(⋅), *TA* also selects a cryptographic hash *H*(⋅):{0, 1}* → [0, *n* − 1] denotes as a secure hash function.
  At the end of setup phase, each party keeps their own private keys, and *CA* preloads with the public parameters {*G*_1_, *q*, *P*, *E*, *p*, *n*, *f*, *G*, *P*_*TA*_, *P*_*CA*_, *h*(⋅), *H*(⋅)}.
***B. User registration*** A user needs to register to become a legitimate taxpayer *TU*_*i*_. First, taxpayer generates a random pseudoidentities(i.e. tax identification number) based on *ECC*, which is unique, then performs the process of registration phase based on self-certified public key.
  - Taxpayer′s real identity *RID*_*i*_ ∈ *G*_1_, each taxpayer randomly selects $k_{i}\in Z_{q}^{*}$ and computes *A*_*i*_ = *k*_*i*_ ⋅ *P*, let $ID_{i}^{1}=h\left(A_{i}\right)$, and $ID_{i}^{2}=RID_{i}⊕h\left(k_{i}\cdot P_{CA}\right)\cdot P$. Taxpayer′s pseudoidentity $ID_{i}=\left(ID_{i}^{1}∥ID_{i}^{2}\right)$, which allows only *CA* to reveal the real identity *RID*_*i*_ of taxpayer. Each taxpayer stores a pseudoidentity *ID*_*i*_, and sends {*ID*_*i*_, *A*_*i*_} to *CA*.
  - *CA* maintain an initially-empty registry list. *CA* randomly selects $k_{i}^{\prime }\in Z_{q}^{*}$ and computers the components of taxpayer′s secret keys by $c_{i}=A_{i}+k_{i}^{\prime }\cdot P,\;e_{i}=h\left(ID_{i}∥c_{i}\right),\;{\bar{x}}_{i}=\left(k_{i}^{\prime }+e_{i}\cdot S_{CA}\right)modq$.
    After that, *CA* sends the value ${\bar{x}}_{i}$ and *c*_*i*_ to taxpayer.
  - Taxpayer computes the private keys $x_{i}=\left({\bar{x}}_{i}+k_{i}\right)modq$, and extracts public key *y*_*i*_ by computing the following equation
    $$\begin{array}{c} y_{i}=c_{i}+e_{i}\cdot P_{CA} \end{array}$$
  - The certified address A is the value *h*(*c*_*i*_).
  - *CA* adds (*ID*_*i*_, *c*_*i*_) to the maintained registry list.
  The correctness of the public key derivation follows.
  $$\begin{array}{cc} x_{i}\cdot P & =c_{i}+e_{i}\cdot P_{CA}\\ & =(k_{i}^{\prime }+k_{i}+e_{i}\cdot S_{CA})\cdot P\\ & =\left(k_{i}^{\prime }+k_{i}\right)\cdot P+e_{i}\cdot P_{CA}\\ & =c_{i}+e_{i}\cdot P_{CA} \end{array}$$
***C. User declaration*** *TU*_*i*_ executes the *SC* in the blockchain and views the corresponding tax return. The tax return *M*_*i*_ ∈ {0, 1}*, which we denote by *M*_*i*_ contains: tax payment amount, tax payment time, $TU_{i}^{\prime }\text{s}$ certified address A, and $TA^{\prime }\text{s}$ address, etc.
  - *TA* maintains an initially-empty users list. *TU*_*i*_ first proves his legal identity to the *TA* by sending his/her public key *y*_*i*_, along with a NIZK(non-interactive zero-knowledge) proof *NIZK*_*i*_ = {(*x*_*i*_):*y*_*i*_ = *x*_*i*_ ⋅ *P*} to prove the knowledge of *x*_*i*_ satisfying *y*_*i*_ = *x*_*i*_ ⋅ *P*, as shown in Fig 2. If the *NIZK*_*i*_ holds, *TA* adds (*ID*_*i*_, *y*_*i*_) to the maintained users list.
  - *TU*_*i*_ signs on the *M*_*i*_, randomly selects *d*_1_ ∈ [0, *n* − 1], then computes *σ*_1_ and *σ*_2_. The specific steps are as follows.
    $$\begin{array}{c} \left\{ \begin{array}{c} R\left(x,y\right)=d_{1}\cdot G\\ \sigma_{1}=f\left(R\right)modn\\ Hash_{i}=H\left(M_{i},ID_{i},T_{i}\right)\\ \sigma_{2}=\left(d_{1}^{-1}\cdot \left(Hash+x_{i}\cdot \sigma_{1}\right)\right)modn \end{array}\right. \end{array}$$
  - *TU*_*i*_ encrypts the *M*_*i*_ with the *P*_*TA*_, randomly selects *d*_2_ ∈ [0, *n* − 1], then computes *C*_1_ = *M*_*i*_ + *d*_2_ ⋅ *P*_*TA*_, *C*_2_ = *d*_2_ ⋅ *P*.
    Where the signature on tax return *M*_*i*_ is *σ*_*i*_ = (*σ*_1_, *σ*_2_), the ciphertext is *C*_*i*_ = (*C*_1_, *C*_2_) and sends (*T*_*i*_, *c*_*i*_, *σ*_*i*_, *C*_*i*_, *NIZK*_*i*_) to *TA*.
***D. Auditing*** *TA* verifies the information received. First check the validity of $TU_{i}^{\prime }\text{s}$ certified address A, then look up $ID_{i}^{\prime }\text{s}$ public key *y*_*i*_ by the maintained list. If it holds, the *ID*_*i*_ is an certified legal taxpayer; otherwise, this step is terminated. *TA* computes as follows.
  - check that
    $$\begin{array}{c} A=h\left(c_{i}\right)\;and\;y_{i}=c_{i}+e_{i}\cdot P_{CA} \end{array}$$
  - decrypt the tax return by computing
    $$\begin{array}{c} M_{i}=C_{1}-S_{TA}\cdot C_{2} \end{array}$$
  - verify
    $$\begin{array}{c} \sigma_{1}\overset{?}{=}f\left(\sigma_{2}^{-1}\cdot \left(Hash_{i}\cdot G+y_{i}\cdot \sigma_{1}\right)\right) \end{array}$$
    If the equation holds, the signature is accepted; otherwise the signature is invalid.
***E. Trace*** *CA* traces the identity of illegal taxpayer with his private key *S*_*CA*_. Consequently, the malicious taxpayer′s real identity can be easily derived by locating the registry list maintained by *CA*.
  - Firstly, *TA* runs the *Auditing* algorithm to verify the given signature. If the signature is invalid, it terminates.
  - After *TA* submits the illegal taxpayer′s *ID*_*i*_ to *CA*, then *CA* computes $RID_{i}=ID_{i}^{2}⊕h\left(A_{i}\cdot S_{CA}\right)\cdot P$ by using his private key.

## Security analysis

In this section, we describe the security analysis of proposed protocol by giving some theorem and security proofs. Theorem 1 are against the anonymity. Theorem 2 targets the identity privacy protection. Theorem 3 and 4 are against the unforgeability, and Theorem 5 are against addresses conditional privacy protection. We shall prove that the proposed schemes can satisfy these security properties and successfully withstand the corresponding attacks.

**Theorem 1.** *The proposed protocol satisfies anonymity if ECDH problem and OWHF assumption holds.*
  **proof.** Assume that $A_{anony}$ is an adversary against the anonymity of the proposed protocol with an non-negligible probability λ in the probabilistic polynomial time, then we can construct an example the *ECDH* problem (*aP*, *bP*, *abP*), where $a,b\in Z_{q}^{*}$, the algorithm $I_{anony}$ simulate the challenger to solve the *ECDH* problem.
  **Setup Phase.** $I_{anony}$ executes the initialization algorithm to generate system public parameters and sends to *A*_*anony*_, *A*_*anony*_ can operate a polynomial-bounded number of the following queries the *register oracle* and *sign oracle* while the $I_{anony}$ returns the corresponding response as follows.
  **Register Phase.** Firstly, $A_{anony}$ randomly selects the identity index *i* ∈ *I*_1_, where *I*_1_ is a group of users, and sends to $I_{anony}$ for registration query. $I_{anony}$ calls the registration algorithm and returns (*ID*_*i*_, *A*_*i*_, *c*_*i*_). $A_{anony}$ could make at most *m* register queries.
  **Hash Query.** *I*_*anony*_ will maintain a hash list, $A_{anony}$ randomly selects *Q*_*i*_ to query the hash oracle $O_{Hash}$. If tuple (*i*, *Q*_*i*_, *Z*_*i*_) exits in the hash list, then *I*_*anony*_ returns the corresponding *Z*_*i*_ as the response result. Otherwise, $I_{anony}$ returns a randomly selected element $Z_{i}\in Z_{q}^{*}$ as a response. Meanwhile, $I_{anony}$ will maintain a hash list and update after each query to ensure identical response to repeated hash queries. $A_{anony}$ could make at most *n* register queries.
  **Challenge Phase.** In the phase, the $A_{anony}$ chooses two taxpayer′ $\left(RID_{i_{0}},RID_{i_{1}}\right)$ to request anonymity challenge. $I_{anony}$ runs register algorithm and randomly selects *j* ∈ {0, 1} to generate corresponding (*ID*_*j*_, *A*_*j*_, *c*_*j*_), then sends $A_{anony}$.
  **Guess Phase.** $A_{anony}$ outputs a bit $j^{\prime }$. Then $I_{anony}$ outputs *j*′ as the answer to its *ECDH* challenge. We note that $I_{anony}$ gets the correct answer in the indistinguishable experiment when $A_{anony}$ wins the anonymity game. The probability of solving the *ECDH* problem with success is $Pr\left[I\left(aP,bP\right)\to abP\right]=Adv_{anony}\left(A_{anony}\right)\cdot Pr\left[j=j^{\prime }\right]\cdot Pr\left[I\left(Z_{j}\right)\right]$.
  If $I_{anony}$ successfully solves the *ECDH* problem, the following conditions need to be met:
  1.
$A_{anony}$ correctly chose *j*′, that is *Pr*[*j* = *j*′] = 1/2;
  2. *Z*_*j*_ satisfies (*j*, *Q*_*j*_, *Z*_*j*_), so $Pr[I\left(Z_{j}\right)]\geq 1/n$. Then
    $$\begin{array}{cc} & Pr[I(aP,bP)\to abP]\\ & =Adv_{anony}\left(A_{anony}\right)\cdot Pr\left[j=j^{\prime }\right]\cdot Pr\left[I\left(Z_{j}\right)\right]\\ & \geq \lambda \cdot (1/2)\cdot (1/n)\\ & =\lambda /2n \end{array}$$
  The advantage of $I_{anony}$ in breaking the *ECDH* is non-negligible, which contradicts the *ECDH* hypothesis, so the scheme satisfies anonymity.
**Theorem 2.** *The proposed protocol satisfies identity privacy preserving if no A can obtain the taxpayer′s secret identity information form the public information.*
  **proof.** We will discuss our security properties in the following two different scenarios.
  **Scenario 1.** This scenario occurs mainly in the registration phase, if the A wants to get the components of the private key from the *CA*, which means he/she needs to deduce the equation ${\bar{x}}_{i}=k_{i}^{\prime }+e_{i}\cdot S_{CA}$. However, there is two unknown values $k_{i}^{\prime }$ and *S*_*CA*_, where $k_{i}^{\prime }$ is randomly and uniformly distributed, and solving the *S*_*CA*_ is comparable to solving the *ECDLP*. Therefore, it is impossible for an adversary to get a portion of the taxpayer′s private key.
  **Scenario 2.** This process occurs mainly between the A and the taxpayer *TU*_*i*_, where $TU_{i}^{\prime }\text{s}$ private key *x*_*i*_. In registration phase, A needs to deduce the equation *y*_*i*_ = *x*_*i*_ ⋅ *P* = *c*_*i*_+ *e*_*i*_ ⋅ *P*_*CA*_, apparently, it is as difficult as breaking *ECDLP* to obtain taxpayer′s private key *x*_*i*_. Even though in the first scenario, the A successfully obtains a portion of the $TU_{i}^{\prime }\text{s}$ private key from the *CA*, $x_{i}={\bar{x}}_{i}+k_{i}$, where $k_{i}\in Z_{q}^{*}$ is uniformly randomly distributed. Therefore, in this scenario, the probability of a successfully attacked adversary is negligible.

The proof of unforgeability in this section can be divided into certificate unforgeabiity and signature unforgeability.

**Theorem 3.** *The proposed protocol satisfies certificate unforgeability if ECDLP assumption holds in Generic Group Model(GGM)* [33]
  **proof.** Inspired by the proof of certificate unforgeability in [34], our proof is shown as follows.
  **Setup Phase.** $I_{cert-unfo}$ generates public parameters by executing setup algorithm. Then it forwards the system public parameters *PP* to $A_{cert-unfo}$.

$A_{cert-unfo}$ can operate a polynomial-bounded number of the following queries and $I_{cert-unfo}$ returns the corresponding response as follows.
  **Register Queries.** In the register phase, $A_{cert-unfo}$ randomly makes register query to the register oracle $O_{reg}$ for the public/private key pair of the taxpayer at index *i*. The $I_{cert-unfo}$ returns (*x*_*i*_, *c*_*i*_) following register algorithm and maintains a list *φ*_*i*_ of coding, let the current query number of its list be *m*. Note that in this case, the verification equation implies that *c* = *φ*(*x* − *e* ⋅ *S*_*CA*_), where *e* = *h*(*ID* ∥ *c*) and *S* = *x* − *e* ⋅ *X*. If *S* is not in the list of oracle queried executed by the algorithm, augment the list by adding *S* = *F*_*m*+1_ at the end, and increase the number of queries *m* to *m* + 1.
  **Hash Queries.** $A_{cert-unfo}$ can query the hash oracle $O_{Hash}$ at any time. $I_{cert-unfo}$ returns a randomly a selected element a response. $I_{cert-unfo}$ maintains a hash list H and updates H after each query to ensure identical response to repeated hash queries.
  **Forge.** Finally, the $A_{cert-unfo}$ forges a corresponding key pair (*c*_*j*_, *x*_*j*_), where (*c*_*j*_, *x*_*j*_) has never been queried. Let *F*_*i*_ be the unique appearance in the list, without loss of generality. From the hardness of *ECDLP*, there does not exist a index *i* such that *F*_*i*_ = *F*_*j*_ *modq*, a random value is returned by the oracle, because *F*_*j*_ represents a query for a new encoding at step *j* when the encoding oracle is called. In other words, the probability of successful forgery, that *σ*(*F*_*j*_) = *c*_*j*_, is negligible. Therefore, no efficient, generic adversary forged successful if given only a polynomial number of queries.
**Theorem 4.** *The proposed protocol satisfies signature unforgeability if OWHF assumption and ECDLP assumption holds.*
  **proof.** Suppose that in polynomial time, an adversary $A_{sig-unfo}$ forges the valid signature of tuple {*M*_*i*_, *ID*_*i*_, *T*_*i*_} with an non-negligible advantage, then we can construct the algorithm $I_{sig-unfo}$ to break the *ECDLP*. Given an example of *ECDLP* question about (*P*, *Q* = *xP*), where $P,Q\in G_{q},x\in Z_{q}^{*}$. $I_{sig-unfo}$ calls adversary $A_{sig-unfo}$ as a subroutine to solve the *ECDLP*.
  **Setup Phase.** $I_{sig-unfo}$ runs the initialization algorithm to generate the system public parameter *PP*, and sends it to $A_{sig-unfo}$. In addition, $I_{sig-unfo}$ initialize two empty lists *L*_*H*_, *L*_*S*_, where *L*_*H*_,*L*_*S*_ respectively represents the query of adversary $A_{sig-unfo}$ to hash oracle $O_{Hash}$ and signature oracle $O_{sig}$.
  **Hash Queries.** $A_{sig-unfo}$ can query the hash oracle $O_{Hash}$ at any time in the form of list {*M*_*i*_, *ID*_*i*_, *T*_*i*_, *}, if the list exits, $I_{sig-unfo}$ returns the form of {*M*_*i*_, *ID*_*i*_, *T*_*i*_, *Hash*_*i*_} to $A_{sig-unfo}$ as the response result. Otherwise, $I_{sig-unfo}$ randomly selects element $Hash_{i}\in Z_{q}^{*}$ as a response, while updates the *L*_*H*_ after each query to ensure the same response to repeated hash queries. $A_{sig-unfo}$ could make at most *m* queries.
  **signature Queries.** $A_{sig-unfo}$ can request that $I_{sig-unfo}$ sign arbitrary messages *M*_*i*_ of his choice, and query the signature oracle $O_{sig}$ to get the form of the list {*M*_*i*_, *ID*_*i*_, *T*_*i*_, *σ*_*i*_}, where *σ*_*i*_ = (*σ*_1_, *σ*_2_). If not exists, $I_{sig-unfo}$ computes the signature of message {*M*_*i*_, *ID*_*i*_, *T*_*i*_} and adds the tuple {*M*_*i*_, *ID*_*i*_, *T*_*i*_, *σ*_*i*_} to the list *L*_*S*_. The reply message needs to meet the following equation:
  $$\begin{array}{c} \sigma_{1}=f\left(\sigma_{2}^{-1}\cdot \left(Hash_{i}\cdot P+y_{i}\cdot \sigma_{1}\right)\right) \end{array}$$
  **Forge.** Adversary $A_{sig-unfo}$ saves the tuple {*M*_*i*_, *ID*_*i*_, *T*_*i*_, *σ*_*i*_} and forges a new signature of known message {*M*_*i*_, *ID*_*i*_, *T*_*i*_}, that is, the $A_{sig-unfo}$ outputs $\left\{M_{i},ID_{i},T_{i},\sigma_{i}^{\prime }\right\}$ in polynomial time. Where the hash value of the message in the two signatures constructed is the same. According to the forked Lemma [35], two signatures need to satisfy:
  $$\begin{array}{c} d_{1}\cdot P=\sigma_{2}^{-1}\cdot P\cdot \left(H\left(M_{i},ID_{i},T_{i}\right)+x_{i}\cdot \sigma_{1}\right) \end{array}$$
  $$\begin{array}{c} d_{1}^{\prime }\cdot P=\sigma_{2}^{-1^{\prime }}\cdot P\cdot \left(H\left(M_{i},ID_{i},T_{i}\right)+x_{i}\cdot \sigma_{1}^{\prime }\right) \end{array}$$
  Through the above formula, we can get
  $$\begin{array}{c} \sigma_{2}\cdot d_{1}-x_{i}\left(\sigma_{1}-\sigma_{1}^{\prime }\right)=\sigma_{2}^{\prime }\cdot d_{1}^{\prime } \end{array}$$

$I_{sig-unfo}$ needs to compute $d_{1}^{\prime }=\left(\sigma_{2}\cdot d_{1}-x_{i}\left(\sigma_{1}-\sigma_{1}^{\prime }\right)\right)/\sigma_{2}^{\prime }modq$, which is equivalent to solving the *ECDLP* problem. Therefore, there is no efficient, generic adversary $A_{sig-unfo}$ that achieves a non-negligible probability of break the signature unforgeability.
**Theorem 5.** *The proposed protocol satisfies conditional privacy preserving if ECDLP assumption holds.*
  **proof.** Here, we elaborate the security properties similar to traceability. Conditional privacy preserving means that honest *TU*_*i*_ are anonymous to everyone, and malicious *TU*_*i*_ are traced by *CA*. During the audit phase, the *TU*′s signature verification fails, the *TA* reports to the *CA* about the malicious *TU*′s identity information.
  Only the *CA* can reveal the real identity of the taxpayer based on the unique tax identification number. The pseudoidentities *ID*_*i*_ consists of $ID_{i}^{1}$ and $ID_{i}^{2}$, where $ID_{i}^{2}=RID_{i}⊕h\left(k_{i}\cdot P_{CA}\right)\cdot P$. No one knows *CA*′ private key *S*_*CA*_, unless the A solves *ECDLP*, so no one except *CA* can decrypt to get the $TU_{i}^{\prime }$ real identity. Whenever the authentication fails, our solution could make his/he identity to be forcibly disclosed to the public in an ingenious way so as to enable the illegal actions can be avoided, thus balancing accountability and anonymity, achieving the security property of conditional privacy.

## Implementation

In this section, we analyze its security advantages and disadvantages of the proposed protocol by comparing with other scheme. In addition, we tested the time cost of each phase in the simulation experiment, which proves its practicability and implementability.

### Safety comparison

In the scheme proposed in [18], *RSA* signatures and group signatures are used to achieve identity anonymity and transaction unforgeability, but the certificate unforgeability is not provided. The scheme proposed in [19] is based on multi-signature to realize the anonymity of users and the unforgeability of transaction, which also does not have the security property of unforgeability of certificates and does not provide traceability. Combined with the security analysis of the previous section, we derive the comparison of the security performance of the three scheme. As shown in Table 1.

**Table 1:** Comparison of scheme security.

scheme	Anonymity	Certificate unforgeability	Signature unforgeability	Traceability
[18]	Yes	No	Yes	Yes
[19]	Yes	No	Yes	No
Our scheme	Yes	Yes	Yes	Yes

### Performance analysis

**Fig 3:**
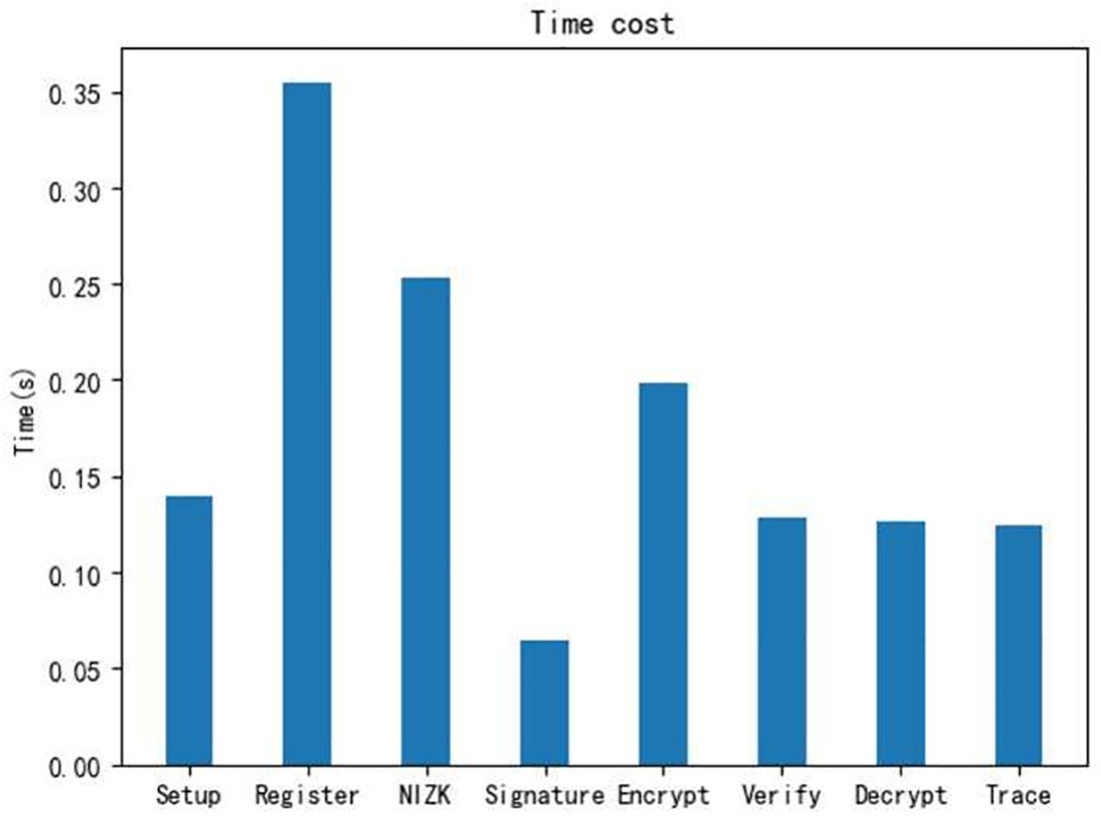
Time consumption of each phase in the single-user.

**Fig 4:**
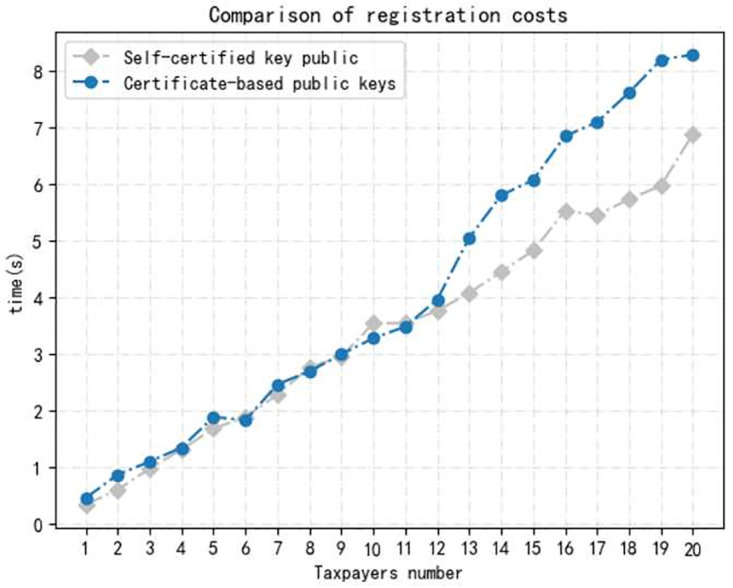
Registration phase comparison.

**Fig 5:**
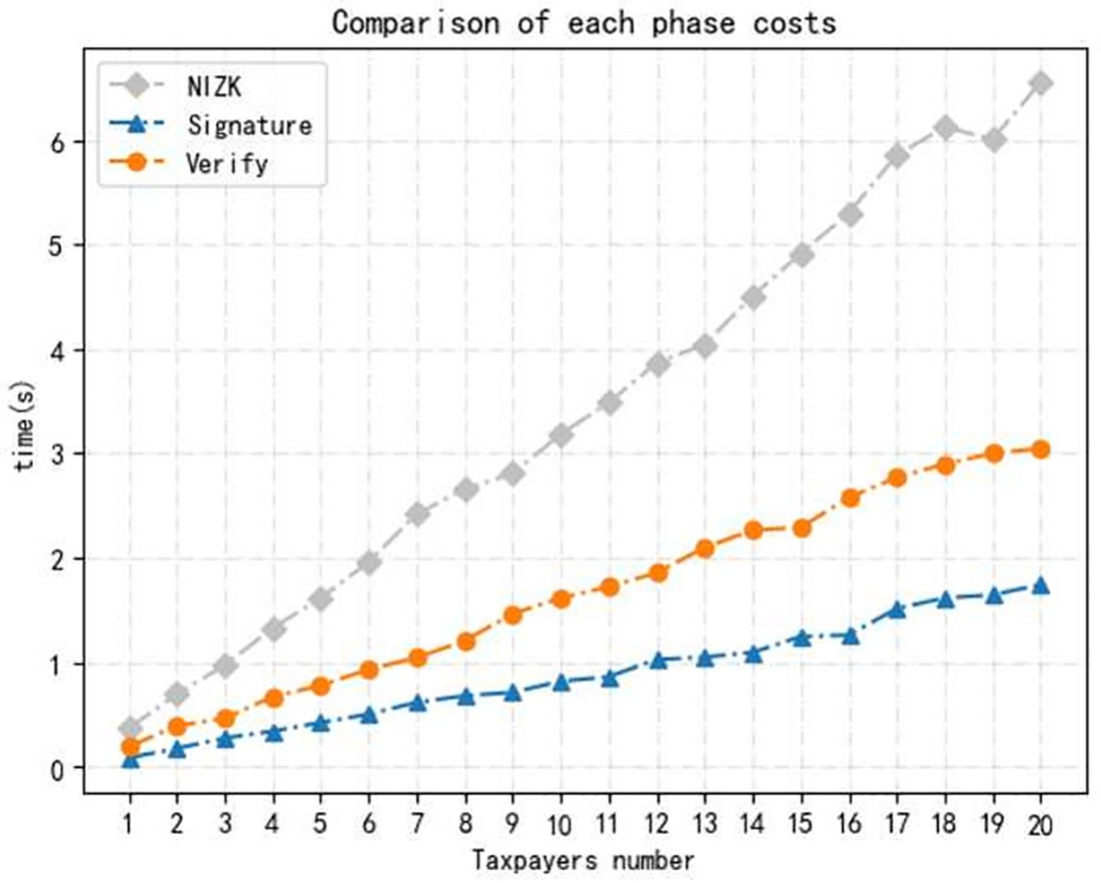
Time consumption of each phase in the multi-user.

**Fig 6:**
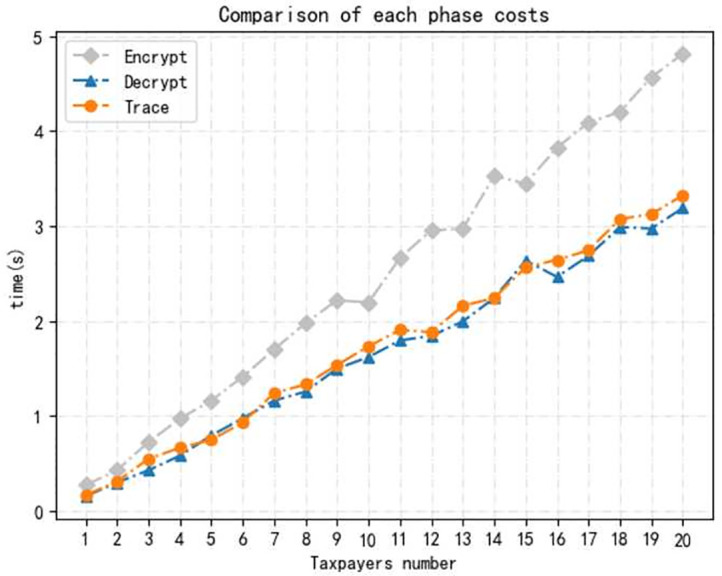
Time consumption of each phase in the multi-user.

1. *Environment.* We conducted the implementation on a desktop loaded with Win 10 operating system and Intel(R) Core(TM) i5–8265U CPU 1.60GHz, 8.00GB RAM. All our evaluations were performed by programs in Python language.
2. We evaluate the time cost of operations in each phase for each taxpayer, including *Setup, Register, Declare, Audit, Trace*, and running 100 times to take an average. As shown in Fig 3. The time cost of Setup is about 0.140*s*, containing system setup, *CA* and *TA* generates his key pair separately. A user who want to become a legal taxpayer has to register, which takes about 0.355*s*, also tested the time costed for *NIZK* proof is 0.253*s*. We evaluate the specific time consumption of each step in declare and audit phase, including signature and verify of the tax return, where the time cost is the 0.064*s* and 0.126s, and the encryption and decryption times were 0.198*s* and 0.128*s* respectively. Finally, the time cost of the tracing phase is 0.124*s*.
3. we increase the number of taxpayers to test time cost. In the registration phase, the time cost of self-certified public key technology and certificate based public key technology are compared, as shown in Fig 4. The results show that the time costs of both increase almost linearly with the number of users, but the self-certified public key technology is more efficient and has better performance in the multi-user case.
4. Testing the time cost of multi-user at each phase. Because the initialization algorithm is executed only once in the whole process, the implementation of the initialization algorithm with multi-user is not considered here. As shown in Figs 5 and 6, where the time consumption of each algorithm tends to increase as the number of users increases. And, in the whole scheme, the zero-knowledge proof algorithm and the encryption algorithm take longer time compared with other phase, but on the whole, the execution time of scheme have higher efficiency.

## Conclusion

In this paper, we propose a blockchain-based certifiable anonymous e-taxing protocol, that guarantees the security requirements of anonymity, unforgeability, and traceability. Our scheme preserve the main merits of elliptic curve cryptography and self-certified public keys, there is no digital certificates, which reduces the reliance on certificate authority, and tax authority can implement implicit verification of certificates while verifying signatures, thus reducing security risks. In addition, the scheme takes advantage of pseudoidentities to achieve conditional privacy, further balancing anonymity and traceability. Finally, we list the security features and some security proofs, the security analysis proves that the scheme has the properties such as anonymity, conditional privacy and unforgeability, etc. Meanwhile, the performance analysis shows that compared with similar schemes, the scheme significantly improves the registration efficiency, proving its practicability and implementability.

## Supporting information

S1 File (ZIP)
